# Future directions for the management of pain in osteoarthritis

**DOI:** 10.2217/ijr.14.10

**Published:** 2014-04

**Authors:** Nidhi Sofat, Anasuya Kuttapitiya

**Affiliations:** 1 Institute of Infection & Immunity, St George’s, University of London, Cranmer Terrace, London, SW17 ORE, UK

**Keywords:** analgesia, bone marrow lesions, cartilage, NSAIDs, opiates, osteoarthritis, pain, quantitative sensory testing, subchondral bone, synovium

## Abstract

Osteoarthritis (OA) is the predominant form of arthritis worldwide, resulting in a high degree of functional impairment and reduced quality of life owing to chronic pain. To date, there are no treatments that are known to modify disease progression of OA in the long term. Current treatments are largely based on the modulation of pain, including NSAIDs, opiates and, more recently, centrally acting pharmacotherapies to avert pain. This review will focus on the rationale for new avenues in pain modulation, including inhibition with anti-NGF antibodies and centrally acting analgesics. The authors also consider the potential for structure modification in cartilage/bone using growth factors and stem cell therapies. The possible mismatch between structural change and pain perception will also be discussed, introducing recent techniques that may assist in improved patient phenotyping of pain subsets in OA. Such developments could help further stratify subgroups and treatments for people with OA in future.

Osteoarthritis (OA) is the most common arthritic joint disorder that is typified by significant structural joint damage, functional impairment and pain [1,2]. There are currently no treatments that are known to modify disease progression. At present, licensed treatments for OA are focused on the relief of pain symptoms and other physical treatments aiming to improve function – that is, physiotherapy and rehabilitation [3]. Many people with OA continue to suffer from pain symptoms despite currently available treatments [4,5]. As the incidence of OA continues to rise in an aging population worldwide, there remains a high unmet need to develop new treatments for OA that target symptom relief and improve patients’ quality of life [6]. Disability in OA arises from pain, reduced range of movement and diminished control of the affected joint. The pain and functional consequences of OA are responsible for the large burden of morbidity in the community. In a study by Hochberg *et al.*, women (but not men) with OA of the knee had higher morbidity and cumulative mortality rates between the ages of 55–74 years [7]. Increased mortality has also been associated with OA of the knee in Sweden [8]. Although comorbidities may result in the increased mortality, it is important to consider the extent to which OA contributes to the deterioration of an individual’s wellbeing. To date, few disease-modifying therapies exist for the treatment of OA. In comparison, inflammatory arthritis, for example, rheumatoid arthritis and psoriatic arthritis, can often be successfully treated with immunomodulatory therapies, including methotrexate and TNF inhibitors, which delay disease progression [9].

This review will highlight areas of recent developments in our understanding of pain in OA. We discuss potential novel therapeutic options for OA pain management, with an evaluation of targets for local mediators in the OA joint, including proinflammatory molecules, neurotransmitters including ion channels, opioids and NGF, together with the modulation of cartilage/bone turnover including agents such as strontium ranelate and bisphosphonates. Local intra-articular therapies for OA could also prove to be effective in future and the authors will discuss the rationale for trials aimed at potential therapies, such as intra-articular FGF-18. Trials are also under way for the use of biological agents including mesenchymal stem cells (MSCs) in the treatment of cartilage defects in OA. While the OA novel treatment pipeline develops, recent work has also focused on optimizing treatment pathways for existing drugs, including NSAIDs, opiates and centrally-acting analgesics, for example, the serotonin–noradrenaline reuptake inhibitor duloxetine in the treatment of OA will also be discussed.

## Pathological changes in the osteoarthritic joint

**Figure 1:**
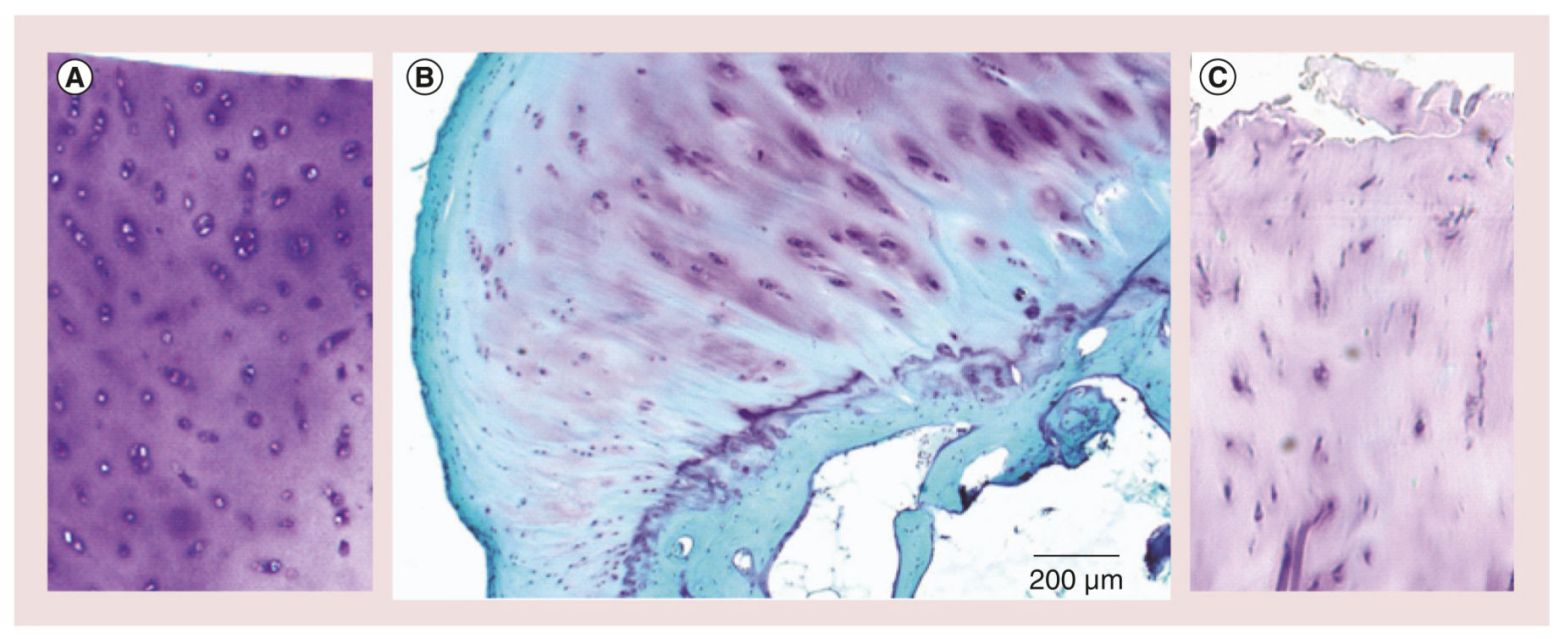
Histological features of tissue damage in osteoarthritis **(A)** There is abundant staining of proteoglycans within cartilage with chondrocytes visible within this section of normal cartilage stained with toluidine blue. **(B)** Early osteoarthritic cartilage showing loss of cartilage extracellular matrix staining, reduction in chondrocytes and early fibrillation of the articular surface of cartilage; stained with fast green and toluidine blue. **(C)** Severely damaged osteoarthritic cartilage showing profound loss of proteoglycans staining and fissuring of the cartilage articular surface. The section is stained with toluidine blue. Osteoarthritis samples **(B)** and **(C)** were obtained at the time of joint replacement surgery from patient with osteoarthritis. Normal cartilage was obtained from a donor undergoing surgery for osteosarcoma. Full informed consent was obtained for all studies.

OA is an arthropathy of synovial joints that is characterized by cartilage loss in which there is often evidence of a periarticular bone response [10]. In the early stages of disease, cartilage develops irregularities at the surface where it becomes fibrillated and appears moderately hypercellular [11]. As the condition progresses, deep clefts form in the cartilage, with loss of aggrecan and type II collagen within the cartilage extracellular matrix (Figure 1). Chondrocytes also clump within cartilage, surrounded by regions of intense staining material indicating increased proteoglycan. As ongoing cartilage damage occurs, the articular joint surface is damaged, leading to loss of joint function. Recent work has shown that cartilage is not the only structure undergoing pathological change in OA, and other important structures in the OA joint, for example, bone marrow lesions (BMLs) [12] and synovitis [13] have an impact on pain perception and OA pathophysiology, which will be discussed in further detail in this article.

Clinically, OA can be divided into a number of subsets. Nodal OA is a well-recognized subset, characterized by polyarticular interphalangeal joint involvement of the fingers. There is formation of Heberden’s nodes (distal interphalangeal joints) and Bouchard’s nodes (proximal interphalangeal joints) [14]. In addition, this subset has a female preponderance, a peak onset in middle age, predisposition to OA of the hip/knee/spine with a marked familial predisposition. OA is a multi-factorial disease in which genetic predisposition, age, estrogen status in women and environmental agents all contribute to susceptibility. In families with hand OA, a greater concordance exists for monozygotic twins than for dizygotic twins [15]. There is also an increased incidence of hand OA in first-degree relatives [16]. Some studies have investigated the nature of the genetic abnormality in subjects with hand OA. Associations have been reported with single nucleotide polymorphisms in the human chromosome 2q that are linked with the IL-1 region on this chromosome [17]. Mutations in an extracellular matrix protein, matrilin-3, have also been linked with hand OA [18]. Several studies have found links between OA and HLA status, including the association of HLA-B, -C, -DR and -DQ in two different studies involving European [19] and Japanese [20] cohorts. Pain severity in OA may also have genetic contributions. A functional polymorphism (Val158Met) in the *COMT* gene is associated with painful knee OA [21]. Other gene polymorphisms involving genes implicated in pain perception, for example, *TRPV1*, have been reported to be associated with painful knee OA [22]. With respect to pain sensitivity, *TRPV1* and the *PACE4* gene *Pcsk6* were associated with pain in knee OA in two separate genetic association studies [23]. Recently, a large consortium genome-wide association studies in 7410 subjects with OA, the arcOGEN study, showed several significant loci relating to cartilage metabolism and obesity [24]. Results showed the most significant association was with the *GLT8D1* gene, associated with glycosylation of cartilage proteins [24]. Other significant associations included the *CHST11* gene, associated with the metabolism of cartilage proteoglycans and the *FTO* gene, which is linked to body weight and obesity. It, therefore, appears that some of the clinically recognized risk factors for OA and mediators of cartilage metabolism are reflected in genetic risk signals, leading to the clinical syndrome of pain and reduced function recognized as OA.

**Figure 2:**
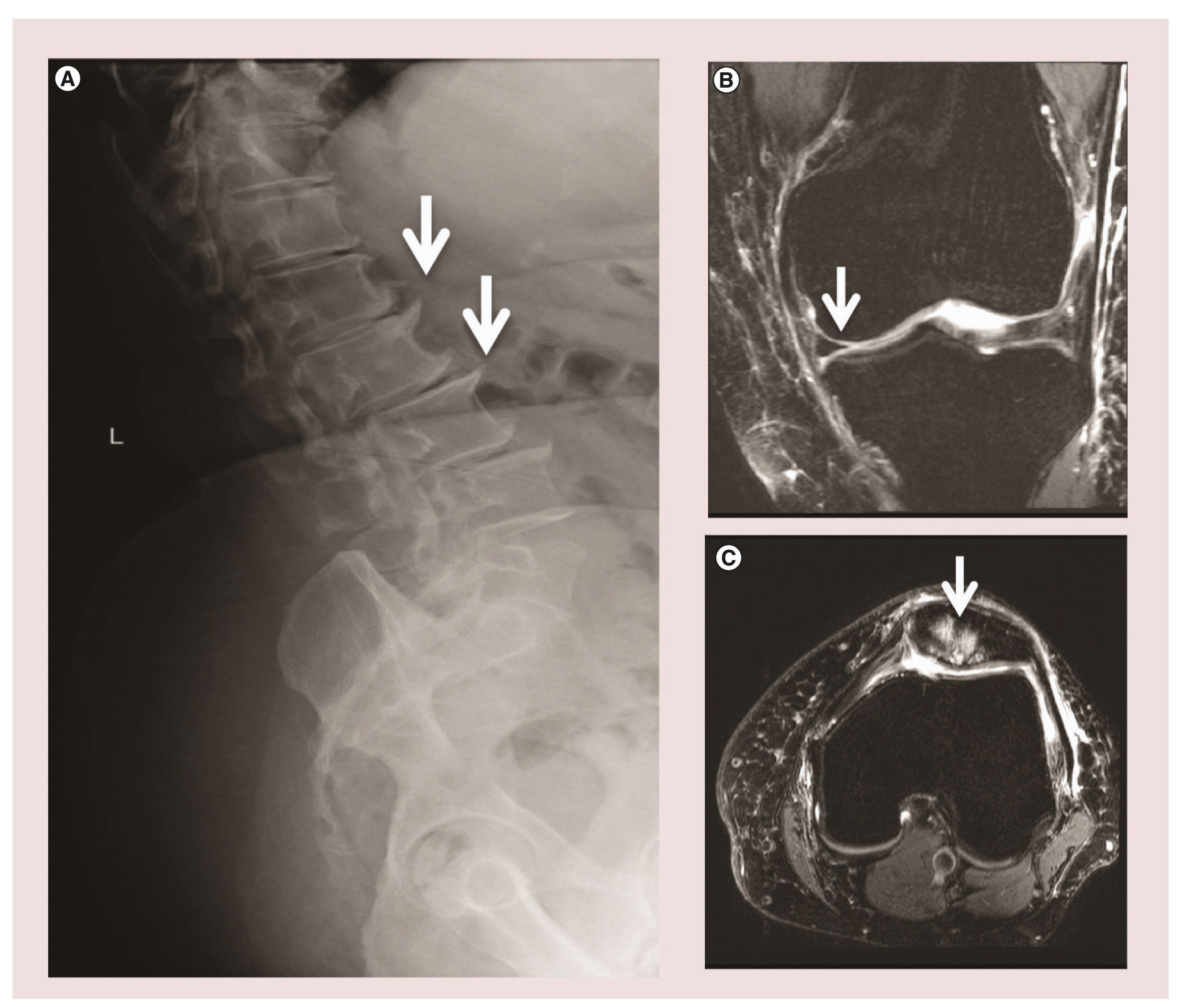
Radiographic features of tissue damage in osteoarthritis **(A)** Example of osteophytes (white arrows) shown in the anterior lumbar vertebral bodies. **(B)** MRI with T2-weighted sequences demonstrating cartilage loss (white arrow) in patient with osteoarthritis. **(C)** MRI with T2-weighted sequences demonstrating bone marrow lesions localized to the knee patella (white arrow) in a patient with osteoarthritis. Image acquisition paradigm for MRIs courtesy of Franklyn Howe (St George’s University, London, UK).

In recent years, there has been a greater understanding of how radiographic changes occurring in the OA joint, including osteophytes, synovitis and BMLs, relate to pain (Figure 2). Typical radiographic features observed by plain radiography, including narrowing of the joint space owing to loss of cartilage, osteophyte formation, bone sclerosis and bone cysts, can be better understood in the context of changes within other joint structures, including synovium and bone, which are aided by MRI techniques [25]. However, it is still unclear as to which changes are most important for pain perception. It has been suggested that BMLs and synovitis have the highest correlations with pain [26,27]. The correlations of pain with synovitis and BMLs will be used as a basis for the discussion of novel therapies for pain in OA in the sections below.

## Risk factor modification for OA

Apart from the genetic associations already described, the development of OA is also linked with other risk factors. Several studies have reported a correlation of obesity with an increased risk of knee OA [28–31]. A Finnish group observed 823 subjects without baseline knee OA in which a strong correlation of incident knee OA with BMI was found (odds ratio: 1.75; 95% CI: 1.0–2.8), with a higher odds ratio (odds ratio: 7.0; 95% CI: 3.5–14.1) for the group with a greater BMI (BMI ≥30.0) [29]. The Framingham study also analyzed 598 knee OA subjects who demonstrated an increased risk of incident knee OA with a higher baseline BMI (odds ratio: 1.6 per 5-unit BMI increase; 95% CI: 1.2–2.2) [28]. The Chingford study found obesity to be a predictor for the development of contralateral OA in women with unilateral OA [32]. Such results supporting the risk of heavier individuals developing OA are important to consider when discussing modifiable risk factors for OA [33]. Weight loss and exercise are popular interventions for OA [34]; how they influence OA progression and pain is further discussed in the following section.

## Exercise & weight loss

In the case of exercise therapy for OA, land-based or water-based exercise and strength training have been subjected to meta-analyses. Four meta-analyses have found there to be small, but clinically relevant short-term benefits of land-based exercise for pain and physical function in knee OA [34–37]. The duration and type of exercise programs included in the meta-analyses varied quite widely, but interventions often comprised a combination of elements, which included strength training, active range of motion exercise and aerobic activities. Although results were favorable in most types of land-based exercise, no specific exercise program appeared to be more favorable [34–37]. Of note, meta-analyses investigating t’ai chi found favorable benefits in improving pain and physical function in people with knee OA [38,39]. With respect to strength training, a meta-analysis and systematic review published in 2011 showed moderate effect sizes for reducing pain and improving physical function compared with controls [34]. Of note, recent data from MOST suggested that people with knee OA had significant levels of knee instability, which was associated with fear of falling, poor balance confidence, activity limitations and reduced physical function [40], which can all have an impact on the level of physical activity achievable by people with OA by exercise interventions [40]. Although there are reports, particularly from animal models, of high physical activity worsening OA lesions [41], clinical studies have been less clear and current guidance recommends exercise for amelioration of pain and improved function in OA.

Recent reports have outlined the rationale for weight reduction in OA in recommendations from both EULAR [42] and OARSI [43]. In 2007, Christensen *et al.* published a meta-analysis and systematic review of weight management in OA [44]. The authors found reductions in pain and physical disability for overweight participants with knee OA after a moderate weight reduction regimen [44]. The authors reported that a weight loss of 5% should be achieved within a 20-week period, that is – 0.25% per week, for the treatment to have efficacy for pain relief and improved function.

## Osteophytes & their effect on OA pain

Osteophytes, sometimes described as osteochondrophytes or chondro-osteophytes, are a classical feature of OA joint pathology (Figure 2), and are found in people with OA and in experimentally induced models. They can appear early in OA, often a precursor to joint space narrowing. Resulting from endochondral ossification at the margins and areas of cartilage loss in OA joints, these structures arise within tissue close to the chondrosynovial junction from progenitor cells. Progenitors may include MSCs residing within the perichondrium and synovium [45,46], suggesting there is a reserve of pluripotent cells receptive to joint injury. By examining osteophytes of distinct developmental stages within patients, a successive pattern of differentiation can be seen [47]. At first progenitor cells at the osteochondral junction are stimulated by growth factors, such as TGF-β and basic FGF, to proliferate [48]. The cells within the chondrophyte undergo chondrogenesis and deposit extracellular matrix proteins, such as aggrecan and glycosaminoglycan. Within the early osteophyte, chondrocytes undergo hypertrophy followed by endochondal ossification, deposition of bone and formation of marrow cavities. Once the mature osteophyte is fully formed, it will integrate with the subchondral bone and the original cartilage [46,49]. Osteophytes are considered to be an adaptive reaction of the joint to mechanical stress and instability. It has been suggested that they may provide a compensatory role to redistribute weight bearing forces and stabilize joints affected by malalignment and OA [48,50,51]. Osteophytes are often removed at the time of joint replacement surgery or cheilectomy procedures, removing the mechanical pressure they apply to surrounding structures. More recent techniques of unicompartmental joint replacement surgery targets areas that may be specifically affected by such lesions and, therefore, have a good impact on pain and joint translocation in the long term [52]. Osteophytes cause joint pain by stretching and compressing nerves and compromising blood flow, possibly causing motor, sensory impairment and faintness, and, in worse cases, impact surrounding tissue and organs [45], while some osteophytes are asymptomatic and could form within healthy individuals. Reports demonstrate that antiresorptive drugs that prevent the formation of cancellous subchondral bone have no effect on the development of osteophytes. Similarly, no inhibition is seen with doxycycline; however, anti-inflammatory drugs, such as glucocorticoids, have an anti-anabolic effect and halt osteophytosis [53–55]. It is evident that the role osteophyte have on pain and function is dependent on their location and disease stage, in end stage OA of larger joints they may act to stabilize the degenerated joint, while osteophytes of the spine are often painful and debilitating [48].

## BMLs & OA pain

Several studies have demonstrated the correlation of BMLs with pain, particularly in large joint arthritis [27,56]. This field has advanced owing to the development of MRI techniques, which have optimized the use of such technologies in visualizing lesions at the bone–cartilage interface. BMLs are often described as diffuse areas of high-density signal in a T2-weighted, fat-saturated MRI or in short tau inversion recovery sequences (Figure 2) [57]. BML patterns on MRI have been described using various methods, some measured using a binary [58] or semiquantitative method (whole-organ MRI score or 0-3 scale) [59,60] for the presence of lesions, several looking at distribution (global and focal cystic) [61], others based classification on lesion location to the lateral and medial condyle [62], while some addressed changes in BML size based on quantitative measurements (maximal diameter or area of lesion) [63,64]. Although changes in BMLs have been analyzed by a number of methods and measurements, this has not significantly affected the general findings [65]. In a study of people with severe hip OA undergoing total hip replacement, Taljanovic *et al.* found that the quantity of BMLs measured by MRI correlated with severity of pain and the number of microfractures observed by histology [66]. This study was relatively small since data were acquired on 19 patients; however, there are now larger clinical data sets observing the relation of BMLs to pain in OA [12,67,68]. The MOST study, which evaluated 570 subjects, found that the severity of BMLs and synovitis were associated with fluctuation of frequent knee pain and pain severity [12]. MOST also showed that of the two types of structural lesions, BMLs were a better predictor of knee pain. In contrast, other groups have not been able to confirm the correlation of pain with BML as strongly as the MOST investigators [68], although larger BMLs have a more significant correlation with pain [69].

With respect to changes in BML over time, Garnero *et al.* evaluated 377 patients with painful knee OA, reporting that within 3 months, BML scores decreased in 37 and increased in 71 patients [70]. Assessing 182 patients with OA at baseline and at 2-year follow-up, Kornaat *et al.* reported that total size of BML changed in 66% of patients, with change in size of individual lesions as 45%, new lesions appeared in 21%, and existing lesions completely disappeared in 10% of patients [71]. The authors concluded from their study that in OA, BMLs are part of a dynamic process and not a constant finding, as opposed to cartilage loss. BMLs are often associated with other MRI features in OA, including subchondral cysts [72], which are a well-defined area of fluid signal on MRI. Several longitudinal investigations have shown that areas of BML are related to subchondral cysts and that BMLs could be an early precystic lesion. Carrino *et al.* suggested that cysts arise from the regions of BML, and signal size of BML changes with cyst development [72]. While others reported that when BMLs and cystic lesions are in close proximity, the direction in which they change is identical [71]; however, not all BMLs will give rise to a cyst. Histologically, a number of pathologies are seen in BMLs, ranging from edema, fibrosis, osteonecrosis, trabecular abnormalities to bony remodeling [73]. At present, the cause(s) for BML development are not certain, but several possibilities have been suggested. Hunter *et al.* proposed that changes in BMLs are in part mediated by limb alignment since medial BMLs occurred mostly in subjects with varus-aligned limbs, and lateral lesions occurred in those with valgus-aligned limbs [74]. It has been suggested that BMLs develop as a result of subchondral bone ischaemia [75], which impairs the exchange of nutrients and oxygen with articular cartilage. Such pathological processes could reduce cartilage integrity and increase the risk of OA development [76–78]. Some hypothesize that BMLs are a result of bony microcontusions leading to necrosis, or increased intra-articular pressure resulting in the extension of synovial fluid into the subchondral bone and proliferation of myxomatous tissue within bone marrow. A similar theory suggest that BMLs may develop if synovial fluid is pumped into subchondral bone marrow through defects in articular cartilage, or from increased stress placed on the subchondral bone owing to overlaying articular cartilage loss – potentially resulting in subchondral microfracture and marrow edema [79]. Felson and colleagues demonstrated BMLs are more likely to be present in painful knees as opposed to nonpainful knees, finding large BMLs in 37% of patients with symptomatic radiographic OA compared with 2% in the asymptomatic patients (p < 0.001) [56], which was confirmed by Sowers *et al.* [68], but not by Kornaat and colleagues [80]. BMLs were also strongly associated to cartilage loss, primarily within areas overlying the lesion [74]. At end stage OA, the joint harbors many pathological features that contribute to arthritic pain. Owing to this coexistence of such defects, it is difficult to determine which single lesion activates and causes pain. Investigators are currently examining how specific MRI changes correlate with clinical features of OA pain in longitudinal studies as this will be helpful in considering avenues for novel therapies [81].

With respect to therapeutic interventions aimed at modulation of BML, recent work has focused on potential use of drug interventions that have previously been utilized to modulate bone density, for example, bisphosphonate drugs. Bisphosphonates are a class of drugs that inhibit osteoclast bone resorption. A recent meta-analysis by our group evaluating studies involving 3832 patients with OA of the hand, hip, knee and spine found that, overall, bisphosphonates showed limited efficacy in analgesia for OA [82]. However, a few studies did show benefit with specific drugs in the class. In the two largest studies that tested the effects of risedronate in knee OA [83,84], our meta-analysis showed no statistically significant difference in pain or functional outcomes assessed by Western Ontario and McMaster Universities OA Index (WOMAC) with risedronate over placebo arms at doses of 5 mg daily, or 15, 35 and 50 mg weekly. The remaining studies, which could not be evaluated by meta-analysis, showed that bisphosphonates reduce pain greater than placebo or nonreatment controls in OA in Asian, European and North American populations when assessed by visual analog scale and WOMAC outcomes. There was heterogeneity across the studies analyzed, with variability in anatomical position of disease, gender studied, route and frequency of drug administration. Specifically, zoledronate has been used in intravenous formulation in a trial of patients with knee OA. Laslett *et al.* compared clinical outcomes between a single infusion of zoledronate (5 mg/100 ml) to a placebo control group [85]. This trial showed significant improvements in pain using the visual analog scale at 6 months, which was the primary end point of this study. The authors also reported a reduction in total BML area of greater magnitude in the zoledronate group compared with placebo after 6 months (−175.7 mm^2^; 95% CI: −327.2 to −24.3) with a nonstatistically significant trend after 12 months (−146.5 mm^2^; 95% CI: −307.5–14.5). With respect to adverse events, the most common was cold or flu symptoms, which was 78% of the 90% total [85]. In other reports, a flare-up of OA pain and inflammation has also been described with zoledronic acid infusion [86]. It is, therefore, possible that drugs targeting bone turnover may be increasingly considered for modulating processes targeting bone turnover in OA; however, further work is required in this area. In more recent work from Nishii *et al.*, 50 participants with symptomatic hip OA were randomized to treatment with alendronate (35 mg/week and 600 mg/day calcium lactate) or a control group (600 mg/day calcium lactate) for 2 years [87]. Alendronate treatment by standard dose for osteoporosis showed clinical efficacy for decreasing pain, but failed to show preventative effects for structural progression of hip OA. Recent data reported from the NIH OA Initiative cohort of subjects with knee OA investigated changes in pain scores in participants taking bisphosphonate therapy [85]. The study reported significant reduction in numeric rating pain within the first 3 years of bisphosphonate use, with reduction in effects by year 4, possibly owing to reduced compliance. A sample size of 55 patients who were bisphosphonate users was studied and, therefore, larger studies would be useful for further evaluation of therapeutic effects.

Recently, a clinical trial has also been published on the use of another bone modulator: strontium ranelate in the treatment of OA [88]. Strontium ranelate is already licensed for use in osteoporosis. Strontium ranelate is a strontium (II) salt of ranelic acid and is known to increase deposition of new bone by osteoblasts and reduce bone resorption by osteoclasts. A recent double-blind, randomized, placebo-controlled trial, investigated its potential efficacy in OA pain. Reginster *et al.* reported outcomes for patients who had moderate OA of the knee, with Kellgren and Lawrence grade 2/3 and joint space width of 2.5-5 mm [88]. Patients were randomized to either strontium ranelate 1 g/day (n = 558), 2 g/day (n = 566) or placebo (n = 559). This study reported that the rate of disease progression measured by joint space narrowing was reduced in the strontium ranelate group at 1 or 2 g daily compared with placebo. The study group also reported greater reduction in WOMAC pain subscore (p = 0.028) and knee pain (p = 0.065) with strontium ranelate 2 g/day after 3 years of treatment. A more recent analysis of the use of strontium ranelate in the same study showed disease-modifying effect of strontium ranelate in a subset of patients from the Phase III knee OA study SEKOIA using quantitative MRI [89]. The authors showed a reduction in BMLs protects against cartilage loss.

In the future, it remains to be seen whether the tolerability of an agent, such as strontium ranelate, would be sustained for more than 5 years, and if taking such a drug for a certain period of term confers chondroprotection and pain relief or whether indefinite use is required. It should also be recognized that patients at risk of developing deep vein thrombosis and myocardial infarction cannot be prescribed strontium ranelate, suggesting that such an agent would require careful screening and monitoring in the OA population.

Further studies have recently been published reporting the use of specific pharmacological agents to target OA pain. These include a study by Esenyel *et al.* in which nasal calcitonin was assessed for the treatment of knee OA [90]. This study of 220 postmenopausal women demonstrated a significant improvement in pain (p < 0.001), stiffness (p < 0.05) and functional level (p < 0.05) after 1 year of treatment. Other emerging studies, albeit in animal models so far, suggested that inhibition of specific proteases, for example, cathepsin K, could be beneficial in OA treatment. For example, Hayami and colleagues reported that a cathepsin K inhibitor was able to reduce cartilage degradation and osteophyte formation in a rabbit model of OA [91]. Cathepsin K inhibition has also been shown to reduce type II collagen degradation in a guinea pig model of OA [92]. Other agents such as parathyroid hormone have also been shown to improve the structure of articular cartilage, but the effect of parathyroid hormone on pain in OA is as yet unknown [93].

## Targeting synovitis to treat OA pain

**Figure 3:**
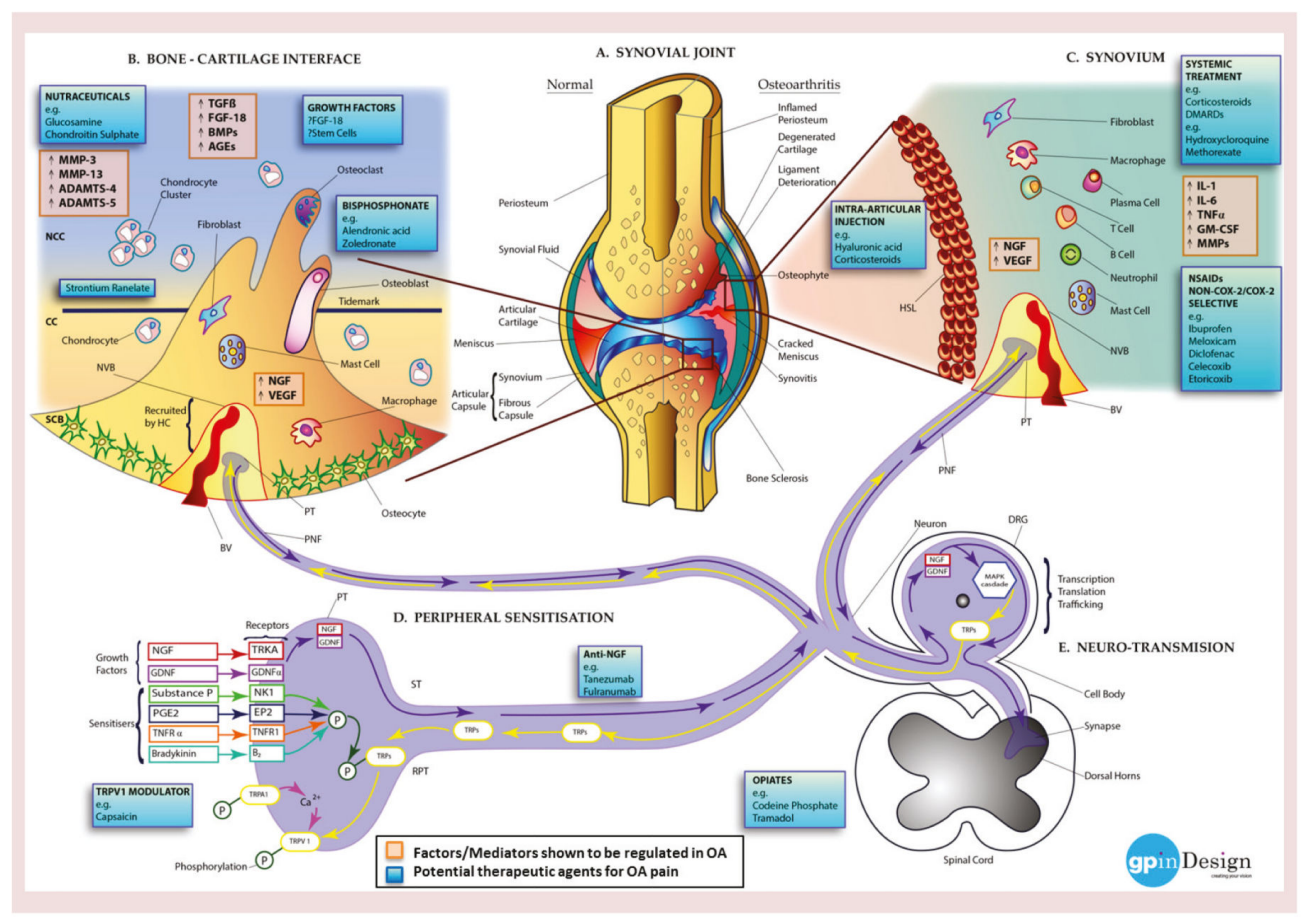
Molecular mechanisms of pain in osteoarthritis (see facing page) **(A)** Left: normal joint structure; right: changes taking place in the synovial joint during the development of OA. **(B)** Bone cells (osteoblasts. osteoclasts and osteocytes), fibroblasts, macrophages and mast cells from the SCB invade the CC and the NCC, via microfractures in the bone–cartilage interface. The disruption of the osteochondral junction promotes production of enzymes, growth factors and molecules (ADAMTS-4, ADAMTS-5, AGEs, BMPs, FGF-18, MMP-3, MMP-13, NGF, TGF-β and VEGF) by the invading cells and chondrocytes stimulating innervation and vascularization ultimately recruiting NVBs (BV and PNF) from the HC. **(C)** Inflammation in the synovium or synovitis causes joint swelling and effusion. Hyperplasia is seen in the synovial tissue resulting in the formation of a HSL followed by the infiltration of inflammatory cells – possibly owing to a systemic response or secondary to cartilage degradation or bone marrow lesion formation. Factors, enzymes and cytokines (GM-CSF, IL-1 family, IL-6 family, MMPs, NGF, TNF-α, and VEGF) stimulated via the cells encourage innervation and angiogenesis (NVB: BV and PNF). **(D)** Peripheral inflammation, cartilage degradation and bone marrow lesions produce numerous inflammatory mediators (e.g., sensitisers: bradykinin, a peptide which causes blood vessels to dilate; E2 stimulates osteoblasts to release factors that stimulate bone resorption by osteoclasts; PGE2; substance P, a neuropeptide belonging to tachykinin neuropeptide family; and TNFR-α) and growth factors (e.g., GDNF and NGF) activating their subsequent receptors (bradykinin receptor B_2_, EP2, GDNF-α, NK1, TNFR1 and TRKA). The sensitizers work by phosphorylating TRPs: TRPA1 and TRPV1, facilitating the trafficking of the channels to the membrane of the PT. Once in the membrane of the PT, TRPA1 modulates calcium exchange with TRPV1 enhancing the nociceptive activity of both channels. The growth factors are transported down the neuron towards the DRG. Altered sensitization of such signaling pathways decreases the pain threshold in OA patients (RPT). **(E)** During ST, growth factors (GDNF and NGF) are transmitted into the cell body of the DRG, where they facilitate intracellular signaling pathways, for example, the MAPK cascade upregulating the expression of TRP channels (TRPA1/TRPV1), which are then transported via the neuron into the PNF and PT. Changes in this pathway during OA can switch the activity of the neurons to an altered state encouraging peripheral sensitization and RPT at the impaired site. Signaling pathways activated in the DRG then take effect in central processes. AGE: Advanced glycation end product; BMP: Bone morphogenetic protein; BV: Blood vessel; CC: Calcified cartilage; DRG: Dorsal root ganglion; EP2: Prostaglandin E2 receptor; GM-CSF: Granulocyte macrophage colony-stimulating factor; HC: Haversian canal; HSL: hyperplastic synovial lining; MMP: Matrix metalloproteinase; NCC: Noncalcified cartilage; NVB: Neurovascular bundle; OA: Osteoarthritis; P: Phosphorylation; PGE2: Prostaglandin E2; PNF: Perivascular nerve fiber; PT: Peripheral terminal; RPT: Reducing the pain threshold; SCB: Subchondral bone; ST: Signal transduction; TRP: Transient receptor potential cation channel. Image courtesy of Gayanthi Perera.

Synovitis is a process characterised by inflammation. It is increasingly recognized that synovitis is a key factor associated with the signs and symptoms of OA, including joint swelling, stiffness and pain [94], which all indicate the presence of synovitis due to a thickened synovium or effusion. Synovitis, which involves the penetration of mononuclear cells into the synovial membrane and the production of prinflammatory cytokines, such as IL-1, IL-6, TNF-α and granulocyte macrophage colony-stimulating factor are upregulated in OA tissue (Figure 3) [95]. There is also increased expression of VEGF and matrix metalloproteinase (MMP) expression in OA synovial tissue, but at significantly lower levels than in patients [94].

Gadolinium-enhanced MRI and ultrasonography are useful and convincing tools for the observation of synovitis [96]. Studies using such methods of imaging suggest that the presence of synovitis may be a marker for the severity and increased risk of the radiographic progression of OA. Systemic high-sensitivity C-reactive protein levels have been reported to mirror synovial inflammation in OA patients and correlate with increased pain [97]. How and why the synovium becomes inflamed during the development of OA has been investigated. One hypothesis is that degraded cartilage fragments, such as advanced glycation end products, contact the synovium: these fragments are recognized as foreign bodies and prompt the synovial cells to produce inflammatory mediators from within the synovium and adjacent cartilage (Figure 3). These mediators are suggested to activate chondrocytes present in the superficial of the cartilage, leading to MMP synthesis and perpetuating cartilage degradation. Such inflammatory mediators may also be involved with synovial angiogenesis and could increase the synthesis of inflammatory cytokines and MMPs by the synovial cells themselves, initiating an irreversible positive feedback cycle [98]. Another theory proposes synovial tissue to be a primary trigger in OA, along with many other cell types involved in many immunological processes have been linked to the initiation and progression of OA [99]. Recently the importance of synovial gene expression to global joint pathology has been supported by the abundance of the synovial fluid proteome with distinct profiles found in healthy individuals compared with early OA in people undergoing arthroscopy after injury of the medial meniscus and late-stage patients undergoing joint replacement [100]. Other findings have also suggested a central role for complement in low-grade inflammation in OA. Proteomic and transcriptomic analyses of synovial fluid and synovial tissue from individuals with OA showed expression and activation of complement in human OA joints [101]. Authors showed that mice genetically deficient in complement component 5 (C5), C6 or the complement regulatory protein CD59a did not develop OA in comparison to their wild-type counterparts in three distinct animal models of OA. The expression of the matrix degrading enzyme MMP-13 colocalized with the complement complex in chondrocytes around osteoarthritic cartilage. It is, therefore, conceivable that molecules targeted to such areas may be of use in the inhibition of cartilage injury in the initial steps during the development of OA.

Synovitis has been targeted with both intra-articular and systemic corticosteroid treatment in previous trials with good effect (Figure 3) [102]. However, the effects of such agents do not appear to be sustained over time. This has led to several researchers calling for the potential need for use of conventional disease-modifying drugs in OA, including methotrexate [103] and hydroxychloroquine [104]. It is interesting to note that corticosteroids in the form of low-dose prednisolone were not shown to be effective in a clinical trial of hand OA [105]. It could therefore be argued that in OA, where low-dose oral corticosteroids are not efficacious, the potential mechanism of disease-modifying anti-rheumatic drugs such as methotrexate and hydroxychloroquine, may be targeted at other compartments apart from synovium, for example, cartilage or bone.

Other groups have argued that more targeted therapies, for example, towards MMP, may be considered. In the largest study of its kind using doxycycline, which inhibits MMP activity, placebo was compared with doxycycline in women with unilateral knee OA [106]. The trial involved treatment with doxycycline 100 mg twice daily in the treatment arm versus placebo, also given twice daily. A total of 431 patients were recruited and showed that after 30 months treatment, doxycycline slowed the rate of joint space narrowing in affected knees. Of interest, drug intake had no effect on joint space narrowing in the contralateral knee, suggesting other factors may also be at play. A recent meta-analysis that included a more recent study showed that doxycycline conferred no overall benefit in pain, with a minimal improvement in joint space narrowing that was outweighed by poor tolerability of the drug owing to side effects [107].

## NSAIDs & nutraceuticals treatment

Traditionally, proinflammatory mediators have been targets for the inhibition of inflammation and consequently pain (Figure 3). NSAIDs inhibit the COX pathway, thereby inhibiting action of prostaglandins and leukotrienes in the OA joint. They are recommended as the first line of treatment for moderate-to-severe OA, used by 20–30% sufferers [108,109], despite the number of individuals who die from NSAID toxicity every year [110,111]. NSAIDs have been one of the most frequently used drugs for over 30 years with 80% of rheumatologists prescribing NSAIDs for symptomatic OA [112–114]. More recently, the second-generation COX-2 inhibitors (rofecoxib, etoricoxib and lumiracoxib) were favored as a safer alternative with superior specificity and efficacy reducing the number of adverse events. However, it was not long before these were also associated with a higher risk of cardiovascular- and gastrointestinal-related adverse events [115,116]. Ultimately, in 2007, the US FDA issued a medication guide for NSAIDs recommending physicians to prescribe the lowest dose for the shortest time possible [117].

Some of the landmark studies of COX-2 inhibitors were conducted in patients with large joint OA; which is especially painful and debilitating [118]. Compared head-to-head, celecoxib and etoricoxib are equally effective in improving pain responses in subjects with hip or knee OA [119]. One of the major issues regarding prescription of NSAIDs is that the population group with OA are often older and may have other significant comorbidity including cardiovascular disease. A meta-analysis of the MEDAL study found that etoricoxib was associated with a higher incidence of hypertension in comparison with diclofenac in people with arthritis [119]. The same meta-analysis suggested that treatment of hypertension with calcium-channel blockers and concurrent NSAID use afforded better control of blood pressure in comparison with other antihypertensive agents assessed.

While NSAIDs provide a short-term relief for OA pain, it is important to consider the long-term effects of anti-inflammatory treatment for a condition primarily initiated by articular cartilage degeneration that can be associated with synovitis. It has been questioned whether there a correlation between the sudden increase in OA: with replacement surgeries between 1997 and 2005 significantly rising: knee replacement’s climbing by 69%, hip replacements by 32% and spinal fusion surgeries increasing by 73% [120], and the widespread use of NSAIDs over the last 30 years. It is also possible that extensive use of NSAIDs and the increase in OA is probably mainly owing to the growing number of elderly and obese individuals. The LINK study tested the effect of indomethacin and tiaprofenic to placebo on radiographic progression of OA in 812 patients [121]. After 1 year of treatment on 376 patients the indomethacin group showed 47% progression of radiographic modifications of OA, while placebo demonstrated only 22%. When comparing this to the tiaprofenic acid group where radiographic progression of OA was similar in both the treatment and placebo group (43 and 34%, respectively), it was concluded that indomethacin accelerated structural damage in OA and this branch of the study was terminated [121]. The majority of reports of NSAID efficacy and tolerability suggests that they do have efficacy for OA pain, particularly in the knee [122,123], but that dosing should be titrated to relative comorbidity and tolerability, with use being focused at times of flare or high symptom severity. At present, guidelines favor the use of topical versus oral NSAIDs if they are efficacious, or oral NSAIDs in severe symptomatic disease for as short a duration as possible [124].

In the quest for novel therapeutic targets for OA pain, several studies in recent years have aimed to compare newer agents to existing therapies for pain. The GAIT trial compared the nutraceuticals glucosamine 1500 mg daily, chondroitin sulphate 1200 mg daily, celecoxib 200 mg daily or placebo in a large randomized trial over 24 weeks [125]. The most rapid response to pain relief was achieved by the celecoxib group, in which the highest number of patients achieved a 20% reduction in the summed score for the pain subscale of the WOMAC index [125]. Although the glucosamine and chondroitin sulfate groups did not achieve superior analgesic relief compared with the celecoxib group in this study of people with knee OA, more recent work has suggested that the nutraceuticals may be of benefit for analgesic relief in a subgroup of patients [126]. Reginster *et al.* also showed improvement in joint space narrowing in people with knee OA treated with glucosamine [127,128]. However, with respect to disease modification, a systematic review has found no statistically significant differences in minimum joint space narrowing between glucosamine and placebo at 1-year follow-up, although a moderate effect was detected at 3 years [129]. Similarly, in the case of chondroitin, four systematic reviews have examined the efficacy of chondroitin for knee OA [129–132]. Results have varied regarding symptom relief, with some reviews finding no significant benefit of chondroitin over placebo and others finding large effect sizes in favor of chondroitin. Results have also been mixed regarding disease modification, with only some studies showing statistically significant decreases in joint space narrowing over a longer 2-year follow-up [129,132].

Other agents targeting glycosaminoglycans turnover in the joint include hyaluronic acid derivatives [133–135]. Hyaluronan is a normal constituent of the synovial joint synthesized by chondrocytes in cartilage and also present in the synovial fluid. It serves to create high viscosity in synovial fluid and buffers fluid loss from joints. A number of formulations have been subjected to clinical trials, including hylan and hyaluronic acid derivatives [133–135]. Most of the trials have been conducted in subjects with painful knee OA. The usual protocol for most of these studies has been repeated injections of hyaluronic acid, for example, series of three injections at weekly intervals. The primary outcome measures included assessment of pain by WOMAC scores. Juni *et al.* showed improvement in pain scores in subjects receiving three different forms of hyaluronan [133]. Of note there, were more adverse effects in the hyaluronan derived from avian sources in comparison with bacterial sources. In this non-industry conducted study, a therapeutic response to pain was maintained even at 6 months. More recent studies have included control arms, for example, hyaluronic acid was superior to saline injection [134] but less effective to corticosteroid injection in the knee [135]. Although a number of studies have described efficacy of hyaluronic acid for pain, especially in knee OA, as outlined above, a recent meta-analysis by Bannuru *et al.* reported no superiority of hyaluronic acid over treatment with NSAIDs [136]. The authors of the meta-analysis did suggest that hyaluronic acid formulations may have some advantages over NSAIDs with respect to safety [136].

## NGF monoclonal antibodies

Since there is a significant side-effect profile associated with long-term use of NSAIDs and opiate analgesics, recent interest in novel pain targets has grown. There has been a focus on NGF as a therapeutic target for pain. In contrast to TNF, NGF acts primarily through a direct action on sensory neurons to induce hyperalgesia. NGF injection into animals leads to prolonged hyperalgesia and allodynia [137]. Increased NGF production has been observed in rheumatoid arthritis and OA synovial cells and chondrocytes [138]. The first clinical trial of a humanized monoclonocal antibody to NGF that binds to and inhibits NGF was published in 2010. In this study, Lane and colleagues reported that 450 patients with knee OA who were randomly assigned to treatment with anti-NGF antibody at 10, 25, 50, 100 and 200 μg/kg bodyweight achieved impressive reductions in walking pain scores measured using the WOMAC index, with a mean of 45–62% reduction with varying doses of tanezumab compared with a placebo response of 22% (p < 0.001) [139]. However, a major concern over this trial was the observation of rapidly progressive OA in a subgroup of such patients and hence the halting of some ongoing trials due to this concern at that time [140]. It has been suggested that the very successful inhibition of the NGF target in some patients could have led to rapidly progressive OA in such cases, and further analysis of this data set is being carried out [141]. Trials of anti-NGF have now resumed and are in progress, for example, tanezumab and fulranumab. More recent studies have also been published to assess the effect of tanezumab in combination with NSAIDs [142] and opioid analgesics [143]. It will, therefore, be interesting to note whether, in a subgroup of patients, particularly those who are not taking NSAID drugs, that anti-NGF inhibition may be a validated therapeutic target in OA.

## Growth factors & stem cell therapy

During development biosynthesis is stimulated by a variety of anabolic cytokines and growth factors, such as TGF-β, bone morphogenetic proteins and FGF. In OA, many factors, such as inflammatory cytokines TNF-α and IL-1, are produced by the synovium and the chondrocytes. In normal adult cartilage, chondrocytes synthesize matrix components very slowly and there is strict regulation of matrix turnover: a delicate balance between synthesis and degradation. In OA, however, this balance is disturbed, with both degradation and synthesis usually enhanced until changes in both bone cells and chondrocytes favor catabolic activity: proinflammatory cytokines, including IL-1, TNF-α and IL-6, act to increase the synthesis of MMPs, decrease MMP enzyme inhibitors and decrease extracellular matrix synthesis. The initiation of such degradative alterations in the joint leads to the depletion of cell reservoirs, loss of the condrogenic potential of cartilage bringing about the preponderance of a fibrogenic phenotype and the structural and functional failure of the joint [144]. Current treatments for cartilage defects in early OA include surgical interventions (microfracture and osteochondral auto/allo-grafts), which have shown promise in clinical trials [145].

Such catabolic changes may have the potential to be reversed by the use of a pool of growth factors [146]. The FGF family of growth factors regulates branching morphogenesis and limb development [147]. FGF-18 is thought to have an anabolic effect on cartilage, leading to increased deposition of FGF-18 in the ribs, trachea, spine and joints. Preclinical data of the anabolic effects of FGF-18 is now being followed-up by Merck Serono in Phase I clinical trials [147]. Investigators are currently looking into the therapeutic potential of endogenous plasma rich in growth factors that may have the potential to modulate gene expression of chondrocytes, synoviocytes, macrophages and MSCs. Therapies involving the utilization of growth factors could have the possibility to stimulate an anabolic microenvironment within an affected joint. A possible approach to maintaining the homeostasis of damaged OA joint tissue could be the use of growth factors, which in turn could improve cartilage/bone dysregulation and lead to reduced pain and improved function [146,148]. Platelet-derived elements, such as platelet-rich plasma, human platelet lysate and platelet supernatants, are carriers of endogenous morphogens, which can be stimulated by endogenous or exogenous activators to modulate cell fate, encouraging cell proliferation and matrix synthesis, alongside anti-inflammatory effects owing to the downregulation of catabolic pathways [148,149]. Platelet-derived elements are convenient and easy to extract, with a high-speed recovery potential offering multiple growth factors at an affordable cost [149]. Platelet-rich plasma injections have had beneficial effects in the treatment of mild-to-moderate OA in approximately 6 months compared with hyaluronic acid and neutral saline injections [148]. Experimental, preclinical and clinical studies are being reported suggesting short-term (1–2 years) improvement, but long-term results on cartilage injuries and joint pain are unknown [149].

MSCs are multipotent precursors of connective tissue cells that can be isolated from a wide variety of adult human tissues, including synovial joints. Endogenous MSCs could possibly act as reservoirs for cell repair or immunomodulatory sentinels reducing inflammation [144]. Current methods rely on the paracrine properties of MSCs that release several growth factors, such as HGF, IGF and TGF, along with anti-inflammatory factors, including cytokines, IL-1ra, indoleamine 2, 3-dioxygenase and HLA antigen-G5 [150]. Chondrocyte and osteoblast phenotypes are established via the activation of pathways induced by paracrine factors, such as the SMAD cascade by BMP-2, TGF-3 or Wnt signaling [151]. Thus, the paracrine factors delivered by the MSCs may be more important for MSC therapeutic potency than stimulating repair responses for the differentiation of cells [144].

Early exploratory research studies used MSC-derived chondrocytes to regenerate cartilage in OA. A hydrated collagen matrix covered with MSCs was implanted into the joint; cartilage regeneration was complete after 6 months, although 20–100% of the new tissue had not integrated into the original cartilage [151,152]. Intervention with local delivery of *ex vivo* cultures of MSCs, as the chondrogenic potential of adult chondrocytes are lost and regression into a fibrotic phenotype initiates, in preclinical models of joint disease has led to promising outcomes and is now being tested in clinical trials recently started in 2013 [144]. Several early-stage clinical trials testing the delivery of MSCs via intra-articular injection into the knee are underway; however, the optimal dose and vehicle are still being optimized [144]. Bader and Macchiarini recently demonstrated the uses of stem cell techniques in several pioneering transplant surgeries, seeding an inert tracheal scaffold with either patient or donor bone marrow MSCs [153]. Further work is needed to characterize factors that could avert MSC derived chondrocyte to undergo premature hypertophy and understand what facilitates terminal development pathways for stable hyaline cartilage regeneration [154]. In the case of both anabolic agents, such as FGF-18, and stem cell therapy trials currently underway, it will be interesting to observe if therapies targeted at regeneration of damaged cartilage in people with OA will translate into improved outcomes for pain and function in the medium to long term.

## Pain sensitization in OA

**Figure 4:**
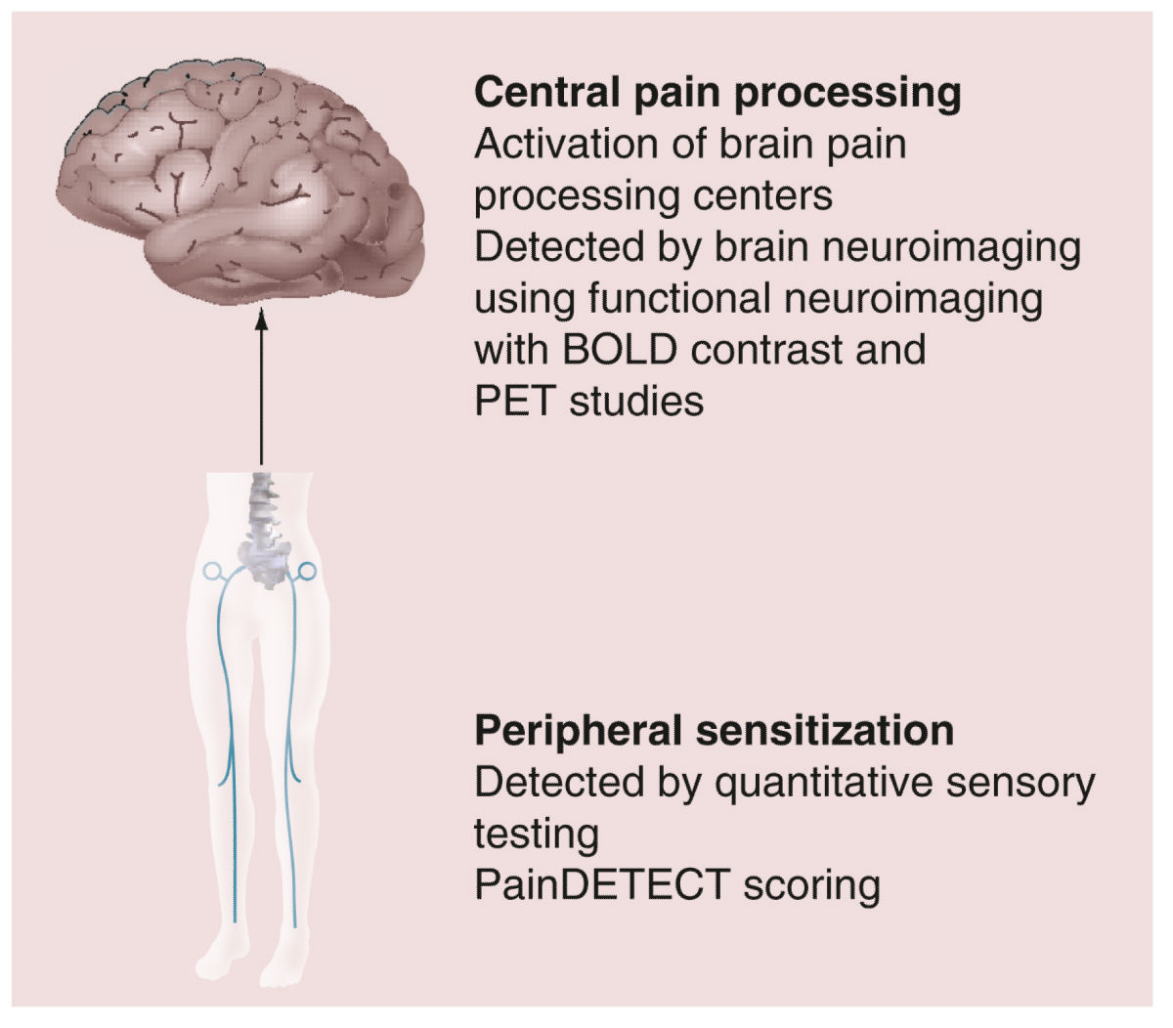
Sensitisation in osteoarthritis Summary of types of studies that have provided information regarding evidence for features of sensitization in osteoarthritis from brain neuroimaging and quantitative sensory testing studies. BOLD: Blood–oxygen level dependent.

In chronic arthritis, a complex set of activation signals lead to the persistence of nociceptive pain. These include known molecular mediators of pain, such as substance P, prostaglandin E2, NGF, TNFR-α, bradykinin, GDNF and TRPV1 (Figure 3). Recent work has focused on tools to measure pain peripherally and centrally in people with OA (Figure 4). Several groups, including work in our unit, have reported the use of quantitative sensory testing in people with OA [155–158]. Pain threshold testing using algometers has become more widely accepted for measuring pain perception objectively since it is reproducible over time and has been validated in large studies with knee OA [159] or intra-oral pain [160]. We have found quantitative sensory testing to be a useful objective measure of hand OA pain [158] where people with hand OA showed evidence of peripheral sensitisation. A recent meta-analysis of pain pressure threshold testing in OA showed that pain pressure thresholds demonstrated good ability to differentiate between people with OA and healthy controls [156]. Lower pain pressure thresholds in people with OA in affected sites may suggest peripheral, and in remote sites central, sensitization. Recent studies have also shown that certain patients with OA may remain sensitized to pain even after joint replacement surgery [161].

Brain neuroimaging tools have also been used to investigate sensitization in OA. Gwilym *et al.* reported increased activation of brain pain processing centers with functional MRI in chronic hip OA, including the thalamus, anterior cingulate and insular cortex, upon quantitative sensory testing [162]. Kulkarni *et al.* reported similar activation using fludeoxyglucose PET in knee OA, suggesting activation of distinct brain regions in patients with chronic arthritic pain [163]. Several authors have described the phenomenon of chronic pain center activation during arthritis as central sensitization, a process thought to derive from hypersensitivity to stimuli by long-term activation of peripheral receptors in arthritic joints. A study by our group in people with hand OA showed significant activation in the thalamus, cingulate and insular cortex but not controls [164]. Of interest, the cingulate cortex is involved in developing emotion formation, learning and memory, suggesting that people with OA are adapting their responses to sensory cues in their hand and developing unique pain activation systems compared with controls. Others have suggested that the cingulate cortex is important in mediating affective processing of pain [165]. With increasing information regarding sensitization in OA, recent trials have reported the use of centrally acting agents, such as the selective serotonin and noradrenaline reuptake inhibitor duloxetine in the treatment of OA [166]. In a recent review, Brown and Boulay discuss the evidence for the efficacy of duloxetine use in four chronic pain conditions including OA [167]. They report that the studies published so far demonstrate a superior analgesic effect of duloxetine compared with placebo that is sustained with continued use and is also safe and effective when used concomitantly with NSAIDs. Further information on the cost utility of duloxetine has shown that it would be cost effective when evaluated in a US population and could be particularly useful in the over 65-year age group when NSAIDs have been prohibitive owing to side effects [168]. Other work by Micca *et al.* has shown that duloxetine is safe in younger and older people with knee OA [169]. Analgesics, such as duloxetine, may have an important role to play as pain-relieving options in patients who are unable to tolerate other classes of drugs or have demonstrated lack to efficacy in response to, for example, NSAIDs and/or opiate drugs.

**Figure 5:**
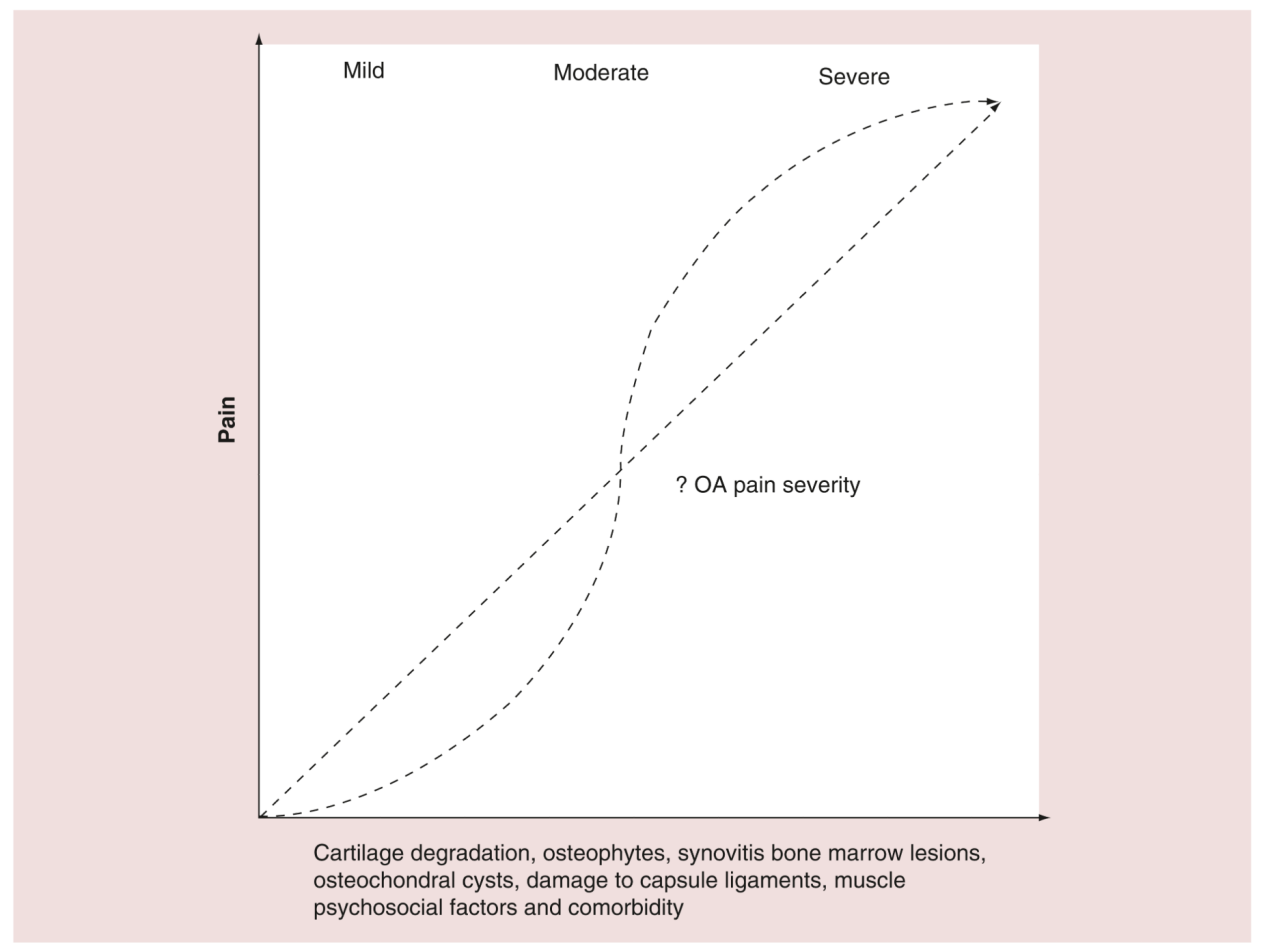
Complex nature of pain in osteoarthritis Graph demonstrating potential contributing factors to OA pain. The multiple trajectories are shown to highlight that the relation between cartilage degradation, osteophytes, synovitis, bone marrow lesions, osteochondral cysts, muscle/ligament damage, psychosocial factors and comorbidity do not always appear to be linearly correlated from emerging studies. OA: Osteoarthritis.

Findings from several large international studies suggest that the correlation between pain and structural change may not be a linear, particularly in a chronic disease, such as OA, when flares may occur (Figure 5). Emerging studies suggest that newer techniques such as quantitative sensory testing and brain neuroimaging may help to further phenotype pain subgroups in OA, which could help to develop pathways for the treatment of OA pain in the future. If it is accepted that pain sensitization is influenced by both physical factors occurring in the joint and psychological influences on pain, then it could be argued that an early combined approach of both pharmacotherapy plus other interventions, such as pain management programs, to inhibit the development of sensitization, for example, before chronic pain develops, could have an effect on clinical pain. Such interventions, early in the disease process, may be effective in modulating the development of chronic pain in OA, but will need to be tested in the context of clinical trials.

## Conclusion

OA is a heterogeneous and debilitating disorder for which there are no universally accepted disease-modifying treatments. It affects large weight-bearing joints including the hip and knee but also smaller joints often in a nodal distribution in the hands. Recognized risk factors include obesity, genetic risk and previous mechanical injury. Since OA is a chronic disease that often progresses after the third or fourth decades, any intervention for pain that is used needs to be safe, with minimal side effects and of long-term benefit. It is interesting to note that many of the agents discussed in this review that could have a therapeutic effect, are also associated with potential harmful effects. For example, NSAIDs, such as indomethacin, can lead to destruction of cartilage, as can treatment with anti-NGF and corticosteroid therapy, suggesting that a positive effect on joint pain may also be associated with accelerated joint destruction, which is an extremely important factor in a chronic, long-term condition such as OA. Recent work highlighted in this review also suggests that the relation between pain and structural damage does not always follow a linear pattern in OA (Figure 5). Recent focus has been on optimizing efficacy of analgesics including NSAIDs and opiates. Emerging data from meta-analyses suggests a limited role for nutraceuticals including glucosamine and chondroitin. The physician looking after OA patients may need to consider the use of centrally acting analgesics, such as duloxetine, if there is lack of efficacy with NSAID/opiates over time and possibly clinical evidence of sensitization. It is only when risk factor reduction, lifestyle advice and pharmacological intervention have been unsuccessful that joint replacement surgery can be considered primarily for OA of the hip and knee.

## Future perspective

Compared with other inflammatory rheumatic diseases, for example, rheumatoid arthritis, there are no disease-modifying treatments for OA. Promising new avenues for understanding the pathophysiology of pain include recognition of NGF as a potential therapeutic target in certain groups with OA, in addition to structure modifying agents including growth factors, such as FGF-18, or stem cell therapies, which are currently in early clinical trials.

## Executive summary

### Pathological changes in the osteoarthritic joint

- Osteoarthritis (OA) is the most common form of arthritis affecting the whole joint, including bone, cartilage and synovium.

### Risk factor modification for OA

- Exercise and weight loss have proven efficacy for OA pain.

### Osteophytes & their effect on OA pain

- Recent work has helped our understanding of how osteophytes form and how they can bring about pain.

### Influence of bone marrow lesions on OA pain

- Larger bone marrow lesions detected on MRI scan correlate with higher OA pain levels, especially in the knee.
- Emerging data is suggesting that structure modifying agents such as zoledronic acid and strontium ranelate could have a disease-modifying effect in OA and improve pain.

### Targeting synovitis to treat OA pain

- Synovitis is often observed by imaging OA joints and correlates with pain.

### NSAIDs & nutraceuticals for treating OA pain

- NSAIDs have proven efficacy to treat OA pain. The main issues with NSAID use are reduced tolerability over time owing to side effects. In large meta-analyses, nutraceuticals do not show significant benefit for pain or structure modification.

### NGF monoclonal antibodies

- Clinical trials targeting molecular mediators of pain, such as anti-NGF, are in progress. Recent published trials, for example, tanezumab in OA, were halted owing to rapidly progressing OA in a subset of patients.

### Growth factors & stem cell therapy

- Cartilage degradation is the hallmark of OA disease and is observed to progress with worsening symptoms. Therapeutic options aimed at regenerating cartilage using growth factors including FGF-18, platelet-rich plasma and mesenchymal stem cells are currently underway.

### Pain sensitization in OA

- Specific subgroups of people with OA show evidence of pain sensitization and future therapeutic avenues could be aimed at modulating this element of OA pain.
